# Supplementary material for: Scaffold-mediated miRNA-155 inhibition promotes regenerative macrophage polarisation leading to anti-inflammatory, angiogenic and neurogenic responses for wound healing
Source: Bioact Mater. 2026 Feb 17;61:373–90. doi: 10.1016/j.bioactmat.2026.02.004 (PMC12926579; doi:10.1016/j.bioactmat.2026.02.004)
Supplement: Multimedia component 1 [file mmc1.docx]

Scaffold-mediated miRNA-155 inhibition promotes regenerative macrophage polarisation leading to anti-inflammatory, angiogenic and neurogenic responses for wound healing – Supporting Information

## **Supplementary Methods**

**Supplementary Method 1.** Haematoxylin and Eosin (H&E) staining of scaffold sections.

A. Deparaffinisation

| **Step** | **Chemical** | **Time** |
| --- | --- | --- |
| **1** | Xylene | 10 min |
| **2** | 100% Ethanol | 5 min |
| **3** | 95% Ethanol | 5 min |
| **4** | 70% Ethanol | 5 min |
| **5** | 50% Ethanol | 5 min |
| **6** | Tap H_2_O | 5 min |

B. Staining

| **Step** | **Chemical** | **Time** |
| --- | --- | --- |
| **1** | Harris Haematoxylin | 5 min |
| **2** | Running Tap H_2_O | 10 min |
| **3** | Acid Alcohol (0.25%HCl in 70% Ethanol) | 3 dips |
| **4** | Tap H_2_O | 5 dips |
| **5** | Eosin | 3 min |

C. Dehydration

| **Step** | **Chemical** | **Time** |
| --- | --- | --- |
| **1** | 95% Ethanol | 5 dips |
| **2** | 100% Ethanol | 5 dips |
| **3** | Xylene | 6 min |

**Supplementary Method 2.** Masson-Goldner staining of scaffold sections.

A. Deparaffinisation

| **Step** | **Chemical** | **Time** |
| --- | --- | --- |
| **1** | Xylene | 10 min |
| **2** | 100% Ethanol | 5 min |
| **3** | 95% Ethanol | 5 min |
| **4** | 70% Ethanol | 5 min |
| **5** | 50% Ethanol | 5 min |
| **6** | Tap H_2_O | 5 min |

**B. Staining**

| **Step** | **Chemical** | **Time** |
| --- | --- | --- |
| **1** | Weigert’s haematoxylin | 5 min |
| **2** | Running H_2_O | 5 min |
| **3** | Acetic Acid 1% | 30 s |
| **4** | Reagent 1 (Azophloxine) | 10 min |
| **5** | Acetic Acid 1% | 30 s |
| **6** | Reagent 2 (Tungstophosphoric acid orange G) | 1 min |
| **7** | Acetic Acid 1% | 30 s |
| **8** | Reagent 3 (Light Green SF) | 2 min |
| **9** | Acetic Acid 1% | 30 s |

**C. Dehydration**

| **Step** | **Chemical** | **Time** |
| --- | --- | --- |
| **1** | 70% Ethanol | 30 s |
| **2** | 95% Ethanol | 30 s |
| **3** | 100% Ethanol | 30 s |
| **4** | Xylene | 5 min |

## **Supplementary Tables**

**Supplementary Table 1.** Lists of antibodies and reagents used for staining and imaging.

| Antibody | Catalogue ID | Supplier |
| --- | --- | --- |
| Anti-CD80 Antibody | [MA5-15512](https://geneglobe.qiagen.com/us/product-groups/quantitect-primer-assays/QT00199367) | ThermoFisher |
| Anti-CD86 Antibody | [MA5-32078](https://geneglobe.qiagen.com/us/product-groups/quantitect-primer-assays/QT01010184) | ThermoFisher |
| Anti-CD206 Antibody | [MA5-32498](https://geneglobe.qiagen.com/us/product-groups/quantitect-primer-assays/QT00041685) | ThermoFisher |
| Anti-CD144 Antibody | 14-1449-82 | ThermoFisher |
| Anti-ICAM Antibody | MA5407 | ThermoFisher |
| Anti-β-tubulin III Antibody | T2200 | Merck |
| Atto-Phalloidin 488 | 49409 | Merck |
| Phalloidin AF 555 | A34055 | ThermoFisher |
| Goat anti-Mouse Secondary Antibody AF 488 | A-11001 | ThermoFisher |
| Goat anti-Mouse Secondary Antibody AF 555 | A-21127 | ThermoFisher |
| Goat anti-Rabbit Secondary Antibody AF 647 | A-21244 | ThermoFisher |
| Hoechst 33342 | 62249 | ThermoFisher |

**Supplementary Table 2.** Lists of genes analysed by qRT-PCR.

| Target Gene | Target Gene Reference | GeneGlobeID |
| --- | --- | --- |
| 18S Ribosomal RNA (18S) | [Hs_RRN18S_1_SG](https://geneglobe.qiagen.com/us/product-groups/quantitect-primer-assays/QT00199367) | QT00199367 |
| Vascular Endothelial Growth Factor (VEGF) | [Hs_VEGFA_1_SG](https://geneglobe.qiagen.com/us/product-groups/quantitect-primer-assays/QT01010184) | QT01010184 |
| Interleukin-10 (IL-10) | [Hs_IL10_1_SG](https://geneglobe.qiagen.com/us/product-groups/quantitect-primer-assays/QT00041685) | QT00041685 |
| Tumour Necrosis Factor-ɑ | [Hs_TNF_1_SG](https://geneglobe.qiagen.com/us/product-groups/quantitect-primer-assays/QT00029162) | QT00029162 |
| Src homology 2 domains containing inositol polyphosphatase 5-phosphatase 1 (SHIP1) | [Hs_INPP5D_1_SG](https://geneglobe.qiagen.com/us/product-groups/quantitect-primer-assays/QT00048370) | QT00048370 |
| B-cell lymphoma 6 (BCL6) | [Hs_BCL6_1_SG](https://geneglobe.qiagen.com/us/product-groups/quantitect-primer-assays/QT00079233) | QT00079233 |
| Suppresor of cytokine signaling 1 (SOCS1) | [Hs_SOCS1_1_SG](https://geneglobe.qiagen.com/us/product-groups/quantitect-primer-assays/QT00202475) | QT00202475 |
| CD80 | [Hs_CD80_1_SG](https://geneglobe.qiagen.com/us/product-groups/quantitect-primer-assays/QT00000497) | QT00000497 |
| CD86 | [Hs_CD86_1SG](https://geneglobe.qiagen.com/us/product-groups/quantitect-primer-assays/QT00033915) | QT00033915 |
| Nitric Oxide Synthase 2 | [Hs_NOS2_1_SG](https://geneglobe.qiagen.com/us/product-groups/quantitect-primer-assays/QT00068740) | QT00068740 |
| Mannose Receptor C-Type 1 (CD206) | [Hs_MRC1_1_SG](https://geneglobe.qiagen.com/us/product-groups/quantitect-primer-assays/QT00012810) | QT00012810 |
| Arginase 1 | [Hs_ARG1_1_SG](https://geneglobe.qiagen.com/us/product-groups/quantitect-primer-assays/QT00068446) | QT00068446 |
| microRNA-155 | [hsa-miR-155-5p](https://www.thermofisher.com/order/genome-database/details/microrna/483064_mir) | [483064_mir](https://www.thermofisher.com/order/genome-database/details/microrna/483064_mir) |

## **Supplementary Figures**

**Supplementary Figure 1. Scaffold-mediated miRNA-155 inhibition is sustained for up to 7 days following scaffold-mediated miRNA-155-i delivery in both M0 and M1 conditions**. A) Scaffold-mediated miRNA-155 inhibition in non-polarised (M0) macrophages. B) Scaffold-mediated miRNA-155 inhibition in pro-inflammatory (M1) macrophages. Data shows mean ± SD, ** indicates p<0.01.

**Supplementary Figure 2. Increasing doses of miRNA-155-i nanoparticles enhance transfection efficiency in both M0 and M1 conditions**. A-C) Delivery of up to 40 pmol of Cy3-tagged miRNA-i nanoparticles does not significantly affect cell viability or number of M0 macrophages. D-E) Increasing nanoparticle dosage leads to enhanced Cy3 expression and transfection efficiency in M0 macrophages. F-H) Delivery of up to 40 pmol of Cy3-tagged miRNA-i nanoparticles does not significantly affect cell viability or number of M1 macrophages. D-E) Increasing nanoparticle dosage leads to enhanced Cy3 expression and transfection efficiency in M1 macrophages. Scale bars = 100 μm. Data shows mean ± SD, *** indicates p<0.001, **** p<0.0001.

**Supplementary Figure 3. CG-155-i scaffolds do not elicit a detrimental response in human dermal fibroblasts**. A-B) Assessment of cell viability through metabolic activity and DNA quantification does not show any significant difference among groups. C) Scaffold-mediated VEGF expression from HDFs does not reveal clear variation for up to 7 days post-transfection. D) Visualisation of cellular morphology displays the distinctive spindle-like morphology of fibroblasts on all scaffold groups. Scale bars = 100 μm. Data shows mean ± SD.

**Supplementary Figure 4. CG-155-i scaffolds support endothelial cell proliferation and the expression of the key endothelial cell surface marker VE-cadherin**. A-B) Analysis of cell viability through metabolic activity and DNA quantification exhibits decreased HUVEC viability on day 3 post-transfection on CG-155-i scaffolds. C) Assessment of VEGF expression does not display any clear differences among scaffold groups. D) Imaging of HUVECs on scaffolds shows a diminished VE-cadherin intensity from HUVECs on CG-Scr-i scaffolds on day 7 post-transfection. E-G) Nuclei count, cell coverage, and VE-Cadherin intensity quantification reveal an improved response from HUVECs on CG-155-i scaffolds while supporting the observation of reduced VE-cadherin expression on CG-Scr-i scaffolds. Scale bars = 100 μm. Data shows mean ± SD, * indicates p<0.05, ** p<0.01, *** p<0.001, **** p<0.0001.

**Supplementary Figure 5. Scaffold-mediated miRNA-155 inhibition does not alter B-cell lymphoma expression in M0 or M1 conditions**. A) BCL6 expression after scaffold-mediated miRNA-155 inhibition in non-polarised (M0) macrophages. B) BCL6 expression after scaffold-mediated miRNA-155 inhibition in pro-inflammatory (M1) macrophages. Data shows mean ± SD.

**Supplementary Figure 6. Secretome from macrophages on CG-155-i scaffolds induces anti-inflammatory responses on endothelial cells.** A) Representative image of LPS-treated endothelial cells used as positive control-E) Endothelial cells exposed to M0 macrophages secretome show a reduced expression of the pro-inflammatory marker ICAM in the CG-155-i group. F-K) The influence of M1 macrophage secretome on endothelial cells exhibits clear morphological changes and decreased ICAM intensity. Scale bars = 100 μm. Data shows mean ± SD, * indicates p<0.05, ** p<0.01, *** p<0.001, **** p<0.0001.

**Supplementary Figure 7. Secretome from macrophages on CG-155-i scaffolds induces faster endothelial cell migration and organisation.** A) Representative images of endothelial cells used for assessment of migration rate following exposure to secretome from macrophages on miRNA-i-activated scaffolds. B) Representative images of endothelial cells used for assessment of vascular-like organisation following exposure to secretome from macrophages on miRNA-i-activated scaffolds. Scale bars = 500 μm
